# Enhanced Transmission at the Zeroth-Order Mode of a Terahertz Fabry–Perot Cavity

**DOI:** 10.1021/acsomega.3c09198

**Published:** 2024-01-04

**Authors:** Soumitra Hazra, Ran Damari, Adina Golombek, Eli Flaxer, Tal Schwartz, Sharly Fleischer

**Affiliations:** † Raymond and Beverly Sackler Faculty of Exact Sciences, School of Chemistry, Tel Aviv University, Tel Aviv 6997801, Israel; ‡ Tel Aviv University Center for Light-Matter Interaction, Tel Aviv 6997801, Israel; § AFEKA—Tel-Aviv Academic College of Engineering, Tel-Aviv 69107, Israel

## Abstract

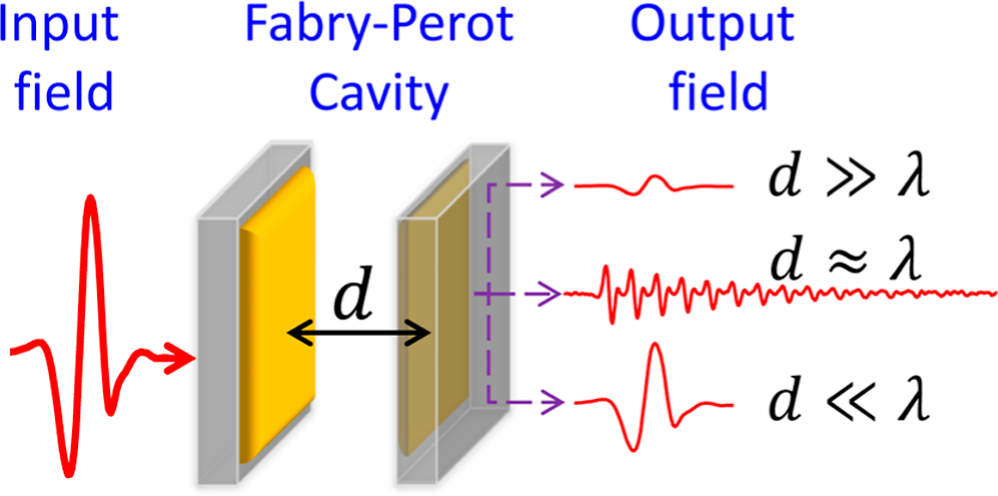

A planar Fabry–Perot cavity with intermirror spacing of *d* ≪ λ is explored for its “zero-order mode” terahertz transmission. The enhanced transmission observed as *d* → 0 indicates that such cavities satisfy the resonance conditions across a broad terahertz bandwidth. The experimental signatures from this elusive, “technically challenging” regime are evidenced using time-domain terahertz spectroscopy and are complemented by numerical calculations. The results raise intriguing possibilities for terahertz field modulation and pave new paths for strong coupling of multiple transition frequencies simultaneously.

## Introduction

The Fabry–Perot interferometer, first introduced by Charles Fabry and Jean-Baptiste Alfred Perot in 1899,^1^ has yielded numerous advancements and applications over more than a century.^2^ From precision wavelength measurements, through atomic line shape analysis and spectral shifts, immense contributions to laser development, sensing, and imaging applications, Fabry and Perot have made a huge impact on all fronts of optical and spectroscopic applications. Recently, Fabry–Perot cavities in the THz frequency range have earned much attention within the study of light-matter interactions in various systems: a two-dimensional electron gas,^3^ superconductors,^4,5^ ferromagnetic nanoparticles,^6^ strongly coupled organic molecules,^7,8^ ro-vibrational polaritons in gas phase molecules,^9^ and many more. The concept of strong light-matter coupling has yielded the novel, highly intriguing field of vibrational strong coupling, where chemical reactions can be manipulated and controlled through zero-point energy fluctuations of the cavity’s optical mode.^10,11^

A Fabry–Perot interferometer (FP) can be constructed from a pair of reflective surfaces (mirrors) positioned parallel to one another at a distance *d* apart. Light traversing the FP undergoes a series of interference events as it bounces back and forth between the mirrors. It is the multiple interferences of the light with itself as it impinges again and again on the cavity mirrors that dictate the transmission and reflection of the incoming light. Constructive interference between these partially transmitted waves at the output of the FP occurs at wavelengths satisfying the relation  at normal incidence, where *m* is an integer. Correspondingly, the condition for resonance of an FP mode is given by , where *f*_*m*_ is the resonance frequency of mode *m*, *c* is the speed of light, and *n* is the refractive index of the medium in the cavity. For many of its applications, the length of the cavity *d* (and thus the FP mode order) is chosen based on practical considerations. For example, in a scanning FP etalon, the accuracy to which a line shape can be determined increases with the mode number, owing to the finite accuracy with which *d* can be set . For strong light-matter coupling experiments, however, *d* is set such that the first FP cavity mode (*m* = 1) frequency is resonant with the transition frequency of the material.^7,10−13^ Thus, in the vast majority of FP applications, the length of the cavity is comparable to or greater than the wavelength of interest.

Previous research on optical phenomena at the deep subwavelength region has focused on harnessing the zeroth-order cavity mode to achieve perfect absorption in ultrathin film absorbers,^14,15^ studying FP resonances at subwavelength cavities formed by sandwiched reflection gratings,^16,17^ and on plasmonic systems such as metallic narrow slits and subwavelength hole arrays.^18,19^ Our current work is aimed at revealing the transmission properties of the zeroth FP cavity mode in a clear and direct way. We monitor the zero-order mode of FP in an open geometry throughout a broad range of intermirror distances, down to *d* ≪ λ. This region possesses technical challenges in the visible or near IR wavelengths as it requires subwavelength precision (≪ 1 μm) over the intermirror spacing. In the terahertz frequency region (THz), characterized by significantly longer wavelengths (1 THz corresponds to 300 μm in wavelength), this requirement is conveniently met. In addition, the THz frequency region enables direct monitoring of the field, *E*(*t*) [rather than the intensity, *I*(*t*)] provided by the electro-optic sampling technique.^20,21^ We utilize time-domain terahertz spectroscopy (TDTS), where a single-cycle THz field traverses the FP cavity and its transmission spectrum is monitored for varying intermirror distances. Our results demonstrate enhanced transmission through the FP cavity as the intermirror distance *d* approaches 0.

## Experimental Section

**Figure 1:**
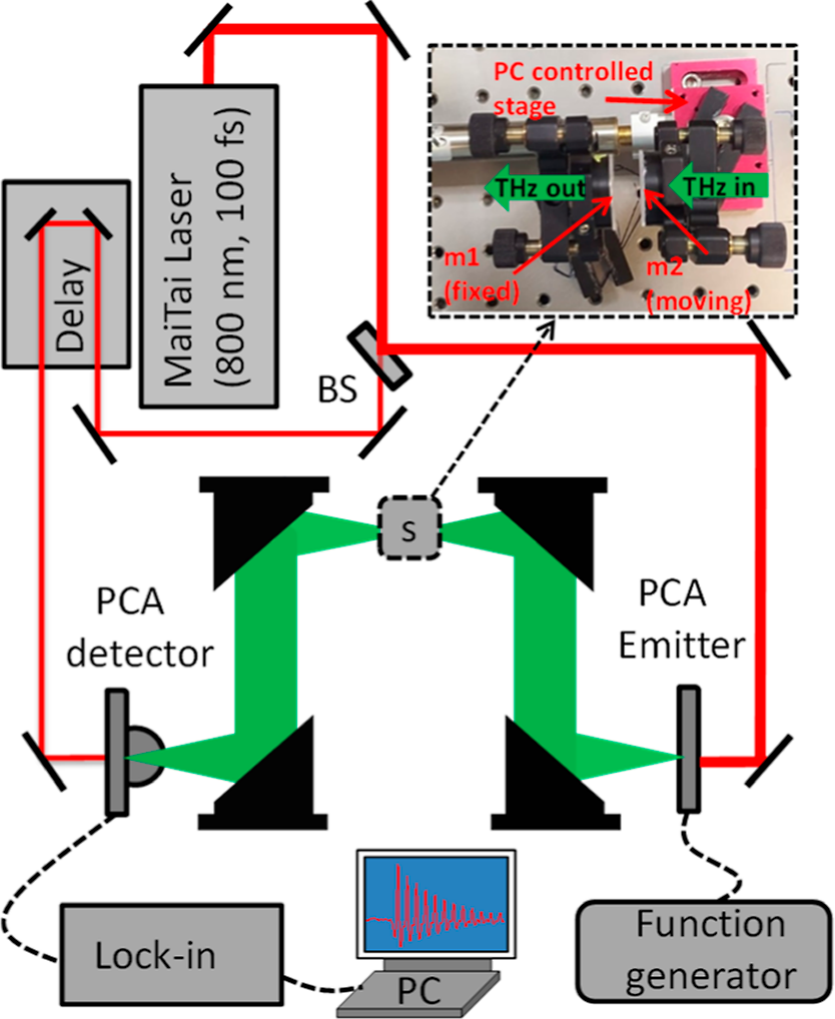
Scheme of the home-built time-domain THz spectrometer setup. The inset shows a photograph of the FP cavity, which consists of a fixed mirror (*m*_1_) and a moveable mirror (*m*_2_).

TDTS measurements were performed in a home-built TDTS setup, pumped by a femtosecond oscillator (Mai-Tai by Spectra-Physics, λ = 800 nm, 100 fs duration). We utilize a pair of photoconductive antennas for THz generation and detection^22^ with a usable bandwidth of 0.15–2.2 THz. The THz field is routed through a 4f optical setup constructed from off-axis parabolic reflectors. The variable-length optical cavity used in this work consists of a movable mirror and a fixed mirror, as in ref (7). The schematic sketch of the time-domain THz spectroscopy setup is shown in Figure 1. A green diode laser is used to align the mirrors parallel to each other by overlapping the multiple reflections from the mirrors in the far field. The cavity mirrors were fabricated by sputtering ∼5 nm of Au on a 1 mm thick fused quartz substrate. While the homogeneity and smoothness of our home-sputtered thin Au films may be compromised and their morphology governed by an island or percolated state, our measurements do not show any spectral ramifications of the latter in the THz reflection (see section S6 in the Supporting Information). The roughness profile as characterized from the atomic force microscopy (AFM) scans is found to be ∼0.6 nm (RMS), i.e., negligibly small with respect to the THz wavelengths used in this work (see section S7 in the Supporting Information). We conclude that our mirrors effectively operate as a smooth and homogeneous layer for our usable THz wavelengths and, in what follows, are characterized by their reflectivity quantified by our TDTS.

## Results

**Figure 2:**
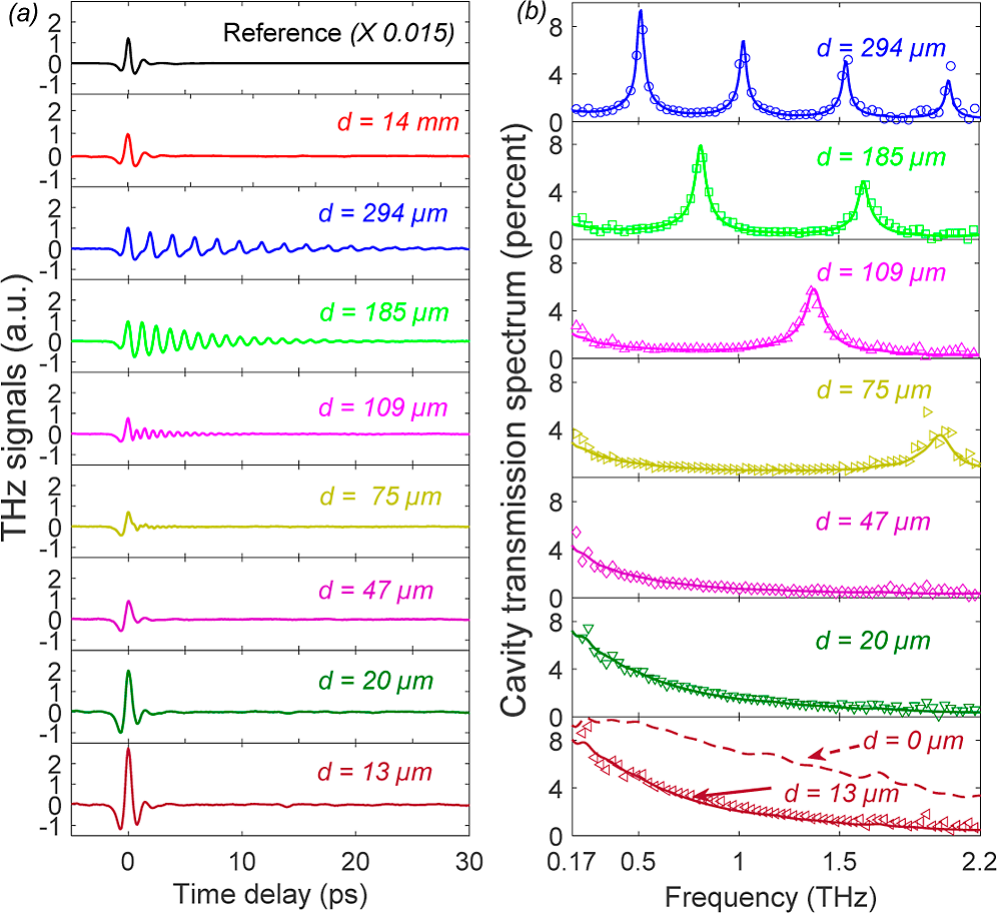
(a) THz transients transmitted through the FP cavity at varying cavity lengths. The incident THz field is measured without the cavity and is marked “reference”. Note that the incident THz field is factored by 0.015 for presentation purposes. (b) THz transmittance spectrum of the FP cavities shown in (a). The empty symbols show the experimental data. The solid lines are theoretical fits (see text).

We conducted a series of TDTS transmission measurements of the FP cavity with varying intermirror distances and obtained a set of THz transients shown in Figure 2a. The incidence time of the THz field was set to *t* = 0 in all of the traces of Figure 2a.

The top panel of Figure 2a depicts the incident THz field and serves as a reference for all of the following transmission spectra. The red curve in Figure 2a shows the THz field transmitted through the FP cavity with *d* = 14 mm and replicates the incident field spectrum only attenuated by ∼80 fold. Note that the round-trip time of the *d* = 14 mm cavity is ∼93 ps—far beyond the 30 ps span of the measurement; hence, it manifests the incident field transmitted through two separated mirrors. For cavity lengths *d* = 294,185,109 μm, the transmitted field exhibits multiple reflections within the time span of the measurement. For *d* = 294 μm we find 4 resonant modes at *f*_1_ = 0.51 THz, *f*_2_ = 1.02 THz, *f*_3_ = 1.53 THz, and *f*_4_ = 2.04 THz that satisfy the relation . These manifest as a beat signal that follows the incident THz field (blue curve in Figure 2a). As the cavity length decreases to 185 and 109 μm, the fundamental resonance frequency increases, leaving two resonant cavity modes and then a single mode, respectively, within the usable THz bandwidth (see Figure 2b).

Note that the peak amplitude of the transmitted field (the very first signal peak at *t* = 0) remains fairly constant in the range *d* = 14 mm → 185 μm. As the cavity length is further decreased, the incident field amplitude seems to decrease in the range of 109–75 μm, after which it gradually increases at *d* < 75 μm, and the ensuing oscillatory signal is practically annihilated. We refer to this region as the zero-cavity mode (*m* = 0), where the intermirror distances are *d* < λ/2, and the first FP mode frequency is well beyond our usable bandwidth. The enhanced THz transmission is quantified by the ratio of the transient THz amplitudes for *d* = 13 μm and *d* = 14 mm, which yields a ∼2.7-fold increase. In what follows, we focus on and analyze this observation.

**Figure 3:**
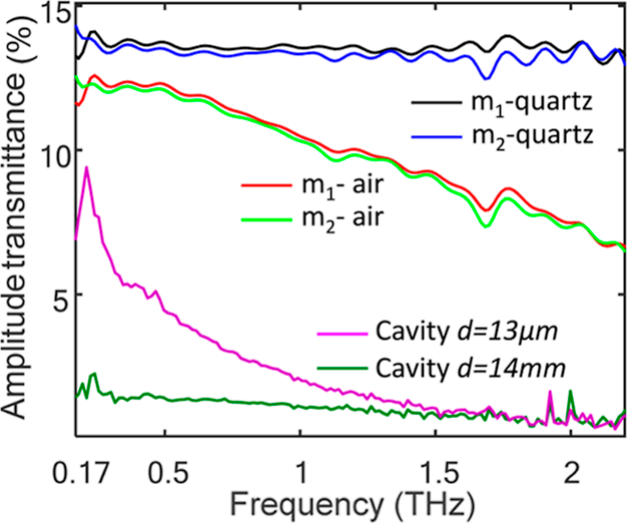
Frequency-resolved THz transmittance for mirrors 1 and 2 quantified with respect to free space (red and pale green, respectively) and with respect to the fused quartz substrate (black and blue, respectively). Transmission spectrum of the FP cavity for *d* = 14 mm (green) and *d* = 13 μm (purple).

Figure 3 depicts the spectral amplitude transmittance of the two cavity mirrors m_1_ and m_2_ referenced against air (red and light green) and against the fused quartz substrate (blue and black), both required for the fitting process discussed later on. The amplitude transmittance through the FP cavity with *d* = 14 mm and *d* = 13 μm is depicted by the purple and green curves, respectively. Each mirror alone (∼6 nm gold deposited on a fused quartz substrate) transmits ∼13% of the THz amplitude across the usable bandwidth, as can be seen from the black and blue curves in Figure 3.

The expected transmission through both mirrors is therefore ∼1.7% (*d* = 14 mm, green curve in Figure 3). For the FP cavity with *d* ≈ 13 μm, however, the amplitude transmittance reaches >9% at the low THz frequency region (0.17–0.2 THz), as depicted by the purple curve, namely 5 fold larger with respect to the “long” (*d* ≫ λ) intermirror length. The gradual decrease in transmission with frequency is clearly observed from the purple curve and will be addressed in the remainder of the paper.

### Numerical Calculations

To calculate the spectra of Figure 2b (solid lines overlaid on the experimental data), we used the analytic transfer function (TF) of the FP cavity for monochromatic light^23,24^
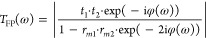
1*t*_1_ and *t*_2_ are the amplitude transmittance of the two cavity mirrors with respect to air as measured by our TDTS setup and shown in Figure 3: *t*_1,2_ = *E*_mirror1,2_(ω)/*E*_air_(ω).

The FP resonance factor is given by 1/(1 – *r*_*m*1_(ω)·*r*_*m*2_(ω)·exp(−2iφ(ω)) where φ(ω) = ω*nd*/*c*) is the phase accumulated through one round trip in the cavity. *n* is the refractive index of the medium inside the cavity (air in this work), and c is the speed of light in vacuum.

The condition for FP resonance requires that the phase accumulated through one round trip is 2π·*m* (where *m* is an integer). For nonabsorbing mirrors, this condition is met at cavity lengths of . Metallic mirrors, however, may inflict an additional phase shift upon reflection due to their absorption loss and manifest as a complex-valued refraction index and corresponding complex reflection coefficient.^25^ Using the complex refractive index of Au thin films of thickness 1.5 and 8 nm reported previously,^26^ we calculated the phase shift upon reflection off the mirrors and found them to vary by up to 3° from π. Furthermore, from the analysis of our experimental data, we obtained an upper limit of  to this reflection phase. We therefore conclude that the additional reflection phase in our experiment is negligibly small and restrict our analysis and discussion to real-valued reflection coefficients in the remainder of this paper. A detailed discussion of the above phase shift is found in sections S4 and S5 of the Supporting Information.

The amplitude reflectance of the mirrors is obtained by fitting eq 1 to the experimental results of several FP cavities with varying lengths ranging from 305 to 93 μm (see Figure S2a in the Supporting Information section S2). This range was selected as it supports multiple resonance modes within our usable THz bandwidth. The cavity length (*d*) used in the fitting process was calculated from the observed resonance peaks that obey: . For simplicity, we associate a single value for the reflectivity of our homemade Au-sputtered mirrors^27^ through our usable THz bandwidth and obtain *r*_*m*1_·*r*_*m*2_ = 0.84 ± 0.01. The latter is the mean reflectivity product of the two mirrors obtained from 23 FP cavities with different lengths. The frequency-dependent amplitude reflection coefficient (*r*_*m*_) measured for the mirrors exhibits a nearly flat response within our usable THz band, as illustrated in Figure S6c in the Supporting Information section. The solid curves in Figure 2b for *d* ≤ 75 μm were calculated using eq 1 with the extracted transmission (*t*_1_, *t*_2_) and reflection *r*_*m*1_·*r*_*m*2_ noted above. The minimal cavity length that we could achieve was *d* = 13 μm due to the imperfect planarity and flatness of the mirror substrates. Note that the experimental transmission at the low THz frequency (∼0.17 THz) tends to coincide with the theoretically calculated transmission for *d* = 0, as depicted by the dashed red curve at the lower panel of Figure 2b.

## Discussion

To explain the observed enhancement in transmission as *d* → 0, we begin by considering the phase accumulated by a field that satisfies the resonance condition of the FP cavity, . The first resonant FP mode (with *m* = 1) is λ = 2*d*, namely, a resonant wavelength of the FP that propagates an exact distance of one wavelength as it traverses back and forth between the cavity mirrors (one round-trip). Considering the optical phase, a resonant field accumulates a phase of 2π + π (or 2π·*m* + π for *m* ≥ 2) throughout each round-trip, where the additional π—phase is independent of d and results purely from the Fresnel reflection off the mirror. This condition sets a constant π-phase difference between the incoming field and the inner cavity field (that propagates an integer number of round-trips in the cavity) as they overlap at the first cavity mirror. Owing to the π phase difference between the two fields at the mirror surface, they impart counteracting forces on the mirror electrons and diminish their field-induced oscillation amplitudes. The latter effectively reduces the reflectivity of the mirror experienced by the incoming field and enhances its penetration into the cavity. This mechanistic description underlies the enhanced transmission of the FP at resonance. Using the above phase considerations, the transmission of the zero-cavity mode (*m* = 0) is readily explained: for a *d* = 0 cavity, the first term in the phase accumulation (2π·*m*) vanishes for all frequency components, leaving only the (frequency-independent) π-phase shift. Correspondingly, the transmission is enhanced at the entire usable bandwidth, as manifested in the enhanced THz field amplitude observed in Figure 2. Imperfections in the parallelism and flatness of the mirrors result in a minimal cavity length of *d* = 13 μm, as noted above. The finite cavity length manifests as decreased transmission with frequency, as observed by the purple curve in Figure 3.

To analyze the transmission of short length cavities, we return to eq 1
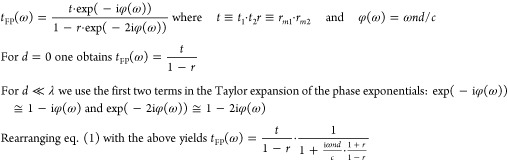
2

**Figure 4:**
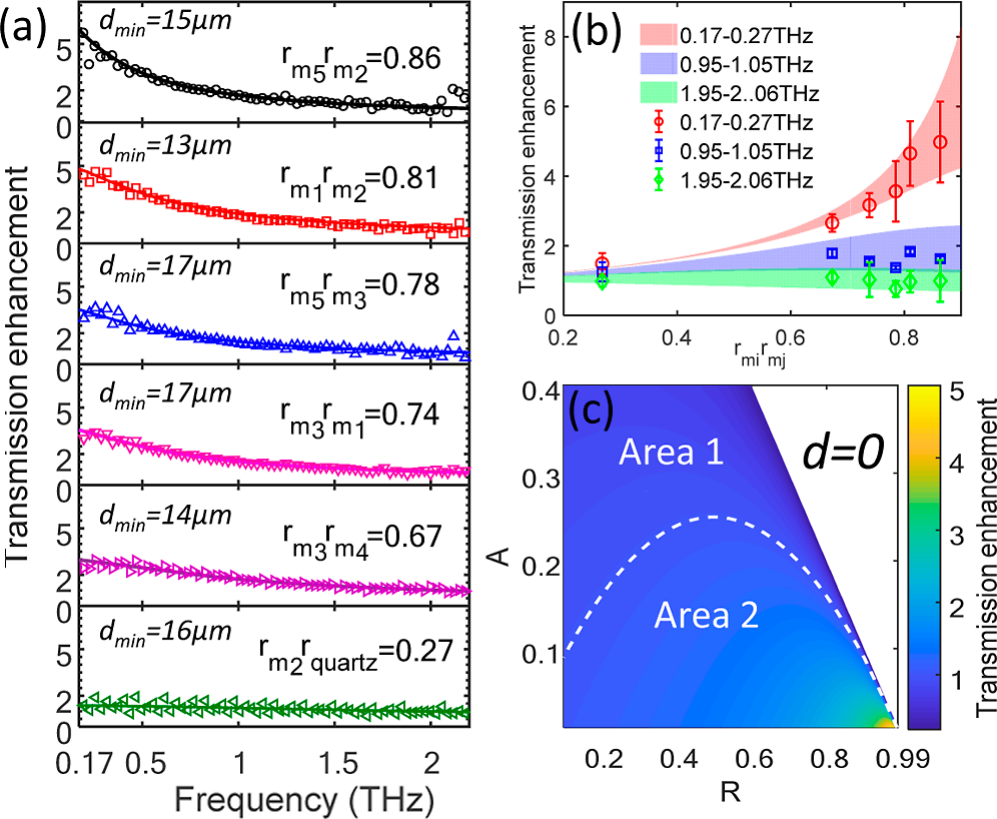
(a) Transmission enhancement of several cavities composed of different mirrors. The enhancement is quantified as the transmission ratio between *d* → 0 (the minimal d obtained is noted in the figure) and *d* = 14 mm. (b) Transmission enhancement vs the reflectivity product of the two mirrors for selected frequency bands. Open symbols depict the experimental data, and pale background areas are simulated for the range of 10 μm < *d* < 20 μm. (c) Theoretical enhancement map as a function of mirror reflectivity (R) and amplitude loss (A) for *d* = 0. The enhanced transmission is quantified with respect to the transmission of a single mirror (see text). The dashed white line shows the equator between area 1 and area 2, where the cavity transmission is lower/higher than the transmission of a single mirror, respectively.

For a given reflectivity *r* and cavity length *d*, the denominator of the second term increases with ω, and the transmission decreases correspondingly. Thus, the transmission at the low THz region (∼0.17 THz) is minimally affected by the finite length of the cavity and is found to be in good agreement with the calculated transmission for *d* = 0. Moreover, eq 2 shows that as the reflectivity of the mirrors increases, so does the “sensitivity” of the cavity to *d* since the phase factor  is factored by , as can be seen from Figure 4a.

Figure 4a depicts the enhancement factor obtained for several mirror pairs differing in Au thickness. We have fabricated mirrors 1–5 on fused quartz substrates with measured transmittance values across our usable bandwidth (0.17–2.2 THz marked respectively): *m*_1_(13.5–13.8%), *m*_2_(13–13.8%), *m*_3_(28–30%), *m*_4_(33–23%), and *m*_5_(8.1–8.3%). We included an untreated fused quartz substrate as a low-reflectivity mirror *m*_quartz_ (88–55%). (For raw transmittance data of the mirrors, see section S1 in the Supporting Information). By pairing the different mirrors from the above cohort, we constructed 6 different FP cavities (*m*_1_–*m*_2_, *m*_3_–*m*_1_, *m*_3_–*m*_4_, *m*_5_–*m*_2_, *m*_5_–*m*_3_, and *m*_2_–*m*_quartz_) and extracted their reflectivity product, *r*_*mi*_·*r*_*mj*_, by fitting to eq 1 (using the same protocol as Figure 2, see Figure S2b in the Supporting Information). The minimal cavity lengths obtained were in the range of 13 μm < *d*_min_ < 17 μm. Figure 4a depicts the enhanced transmission for the different cavities across the THz bandwidth. The enhancement in transmission was experimentally obtained by  (open symbols) and calculated by  (solid curves), where *m*_*i*_ and *m*_*j*_ are indexes for the different mirrors. The results in Figure 4a show that the enhancement factor increases with the reflectivity of the FP mirrors. Figure 4b depicts the measured enhancement for three representative frequency bands (0.17–0.27 THz, 0.95–1.05 THz, and 1.95–2.05 THz). The filled areas (color coded in red, blue, and green, respectively) show the simulated enhancement for cavity lengths 10 μm < *d* < 20 μm. The low frequency band of ∼0.2 THz best represents the enhancement expected for *d* → 0 owing to its “long” wavelength. The enhancement obtained for higher frequency bands (∼1 and ∼2 THz depicted by the blue and green curves, respectively) is strongly compromised due to the deviation of *d* from 0.

Inspired by the enhanced transmission observed as *d* → 0, we set out to explore the breadth of the effect. Different from the above, where the enhancement is quantified by the ratio of FP with *d* → 0 and of the FP with (effective) *d* → ∞, here we focus on a more refined quantity, the transmission ratio between the FP with *d* = 0 and a single mirror. Using the results of Figure 3, we quantify this ratio as ∼0.8 for the low frequency component (∼0.17 THz), namely, the experimental transmission of the FP remains lower than that of a single mirror. Figure 4c shows a theoretical prediction for this ratio for varying mirror parameters. For simplicity, we consider a FP cavity composed of two identical mirrors with amplitude reflectance *r*_1_ = *r*_2_ = *r*, amplitude transmission *t*_1_ = *t*_2_ = *t*, and attenuation loss (A). These three parameters are bound by the conservation relation: . Thus, for a given *A* and *r*, we extract the mirror transmission *t* and plot the ratio of the FP cavity transmission (T) and that of the mirror, given by . For high reflectivity (*r* = 0.98) and low attenuation loss (*A* = 0.01), we predict a ∼5 fold enhancement (). This simplified calculation predicts that for mirror parameters within area II (marked in Figure 4c), where the attenuation loss is sufficiently low and the mirror reflectance sufficiently high—one can increase the transmission through a single mirror by a few-fold by merely placing another identical mirror next to the first. Note that the above calculation assumes a zero distance between the FP mirrors (*d* = 0). Since the practical flatness of any mirror is finite, *d* = 0 is beyond experimental reach. However, tuning the cavity length to *d* ≈ 1 μm or even smaller is possible.^28^ Sufficiently short cavity length is expected to manifest as enhanced transmission without significant compromise to the spectral content (Supporting Information section S3).

## Conclusions

The zero-order mode of a Fabry–Perot cavity manifests enhanced transmission across a broad THz bandwidth. This enhanced transmission suggests that the zero-order mode satisfies the resonance condition for the entire spectrum. The latter may offer uniquely intriguing possibilities in the realm of strong-coupling phenomena, e.g., the simultaneous coupling of multiple transitions of different frequencies within the same cavity. Technical challenges in accessing the zero-order regime are significantly eased by the long wavelength of the THz field, which enables direct experimental exploration of this typically elusive region.

## Data Availability

The data underlying this study is provided within the manuscript and Supporting Information. The raw data is available from the corresponding author upon reasonable request.

## Supporting Information Available

Transmittance curves of the different mirrors used in this research, extraction of amplitude reflectance, FP transmission enhancement calculations, experimentally extracted reflection parameters at varying FP lengths, phase shift at the reflection of the air-mirror interface, upper limit of the phase distortion from cavity transmission measurements, THz reflection measurements, and AFM image of a representative mirror (PDF)
