# Supplementary material for: Enhanced Transmission at the Zeroth-Order Mode of a Terahertz Fabry–Perot Cavity
Source: ACS Omega. 2024 Jan 4;9(2):3000–5. doi: 10.1021/acsomega.3c09198 (PMC10795109; doi:10.1021/acsomega.3c09198)
Supplement: Supplementary file 1 — ao3c09198_si_001.pdf [file ao3c09198_si_001.pdf]

Supporting Information

Enhanced Transmission at the Zeroth-Order Mode of a Terahertz Fabry-Perot Cavity

Soumitra Hazra[1,2†], Ran Damari[1,2], Adina Golombek[1,2], Eli Flaxer[1,3], Tal Schwartz[1,2], and Sharly Fleischer*[1,2,‡]

[1]Raymond and Beverly Sackler Faculty of Exact Sciences, School of Chemistry, Tel Aviv University, Tel Aviv 6997801, Israel
[2]Tel Aviv University Center for Light-Matter Interaction, Tel Aviv 6997801, Israel
[3]AFEKA – Tel-Aviv Academic College of Engineering, 69107 Tel-Aviv, Israel
Email: †soumitra.hazra89@gmail.com, ‡sharlyf@tauex.tau.ac.il

Section S1: Amplitude transmittance of the mirrors and reference THz spectrum

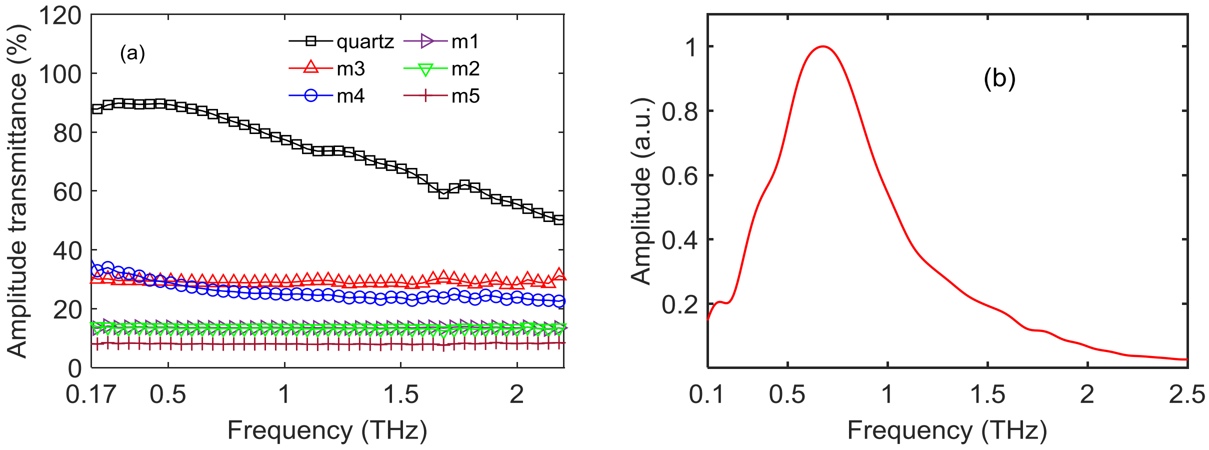

Figure S1: (a) The THz amplitude transmittance through different mirrors (m1, m2, m3, m4, m5) and fused quartz. (b) Reference THz spectrum measured in air.

Section S2: Extraction of amplitude reflectance via fitting to cavity transmission

Fig. S2(a) depicts the FP cavity transmission at varying lengths (d) in the range 305μm→ 109μm. By fitting to eq.1 in the main text file, we extract the reflectivity product of mirrors m_1 and m_2. Figure S2(b) represents the transmission of the FP cavity for the cavity lengths 595μm, 448μm, 248μm, 555 μm, 262μm and 320μm for 6 mirror pairs: m_5-m_2, m_1-m_2, m_5-m_3, m_3-m_1, m_3-m_4, and m_2-quartz respectively. We extracted the reflectivity products of the above mirror pairs ($r_{mi}r_{mj}$) by fitting the cavity transmission to eq.1.

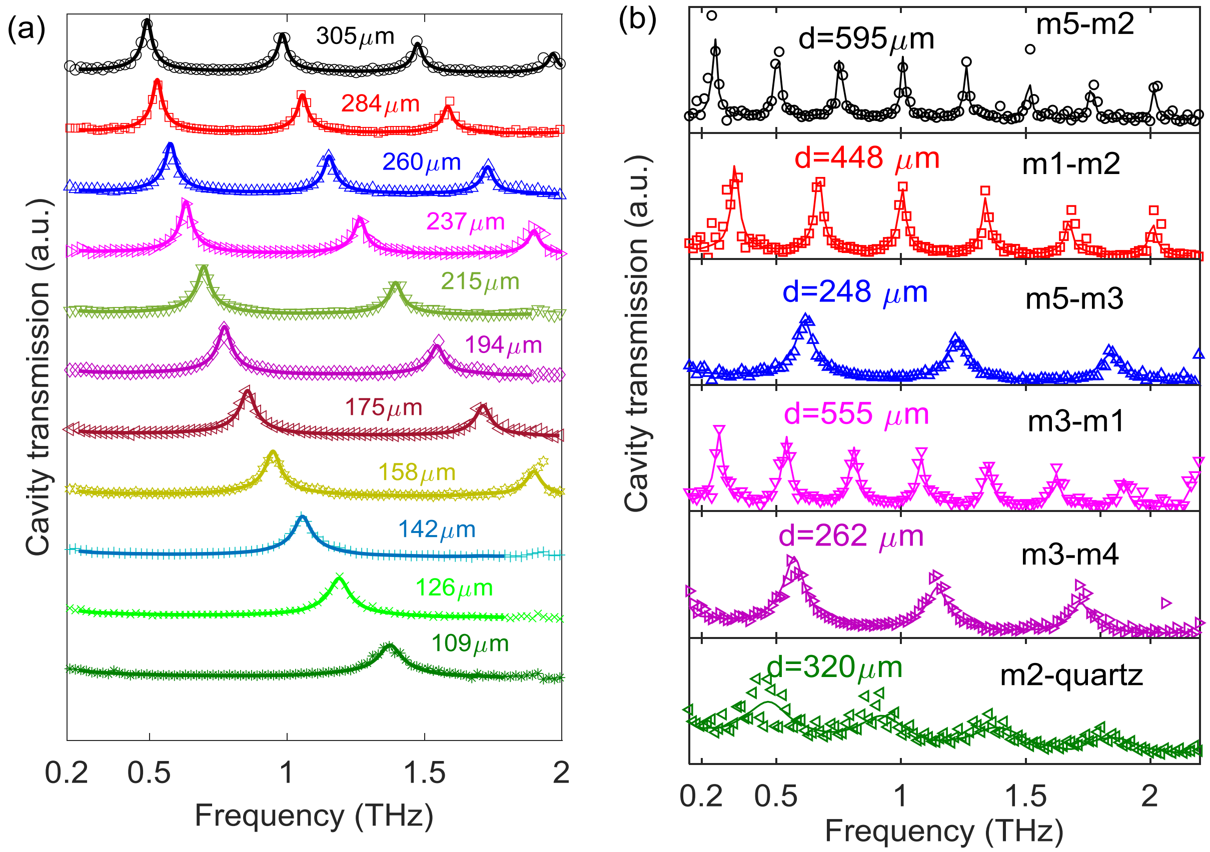

Figure S2: (a) Cavity amplitude transmittance fitted with the FP transfer function (eq. (1) in the main text) for different cavity lengths of FP cavity (m1-m2) **(b)** The cavity transmission for six mirror pairs: m5-m2, m1-m2, m5-m3, m3-m1, m3-m4, and m2-quartz with the cavity lengths 595μm, 448μm, 248μm, 555 μm, 262μm and 320μm respectively. The experimental data is given by the open symbols and the theoretical fit to eq.1 is depicted by the solid lines.

Section S3: Transmission enhancement of FP cavity at $d \neq 0\mu m$ compared to a single mirror transmission

Transmission amplitude of the FP calculated for several cavity lengths using Eq. (1). The cavity is composed of two identical mirrors with parameters R=0.85, A=0.1. The black line depicts the amplitude transmission of a single mirror and the colored lines show the FP transmission at cavity lengths $(0\mu m \leq d \leq 2\ \mu m)$. As noted in the text, we predict enhanced transmission for finite cavity lengths with respect to the transmission of a single mirror. Reduction in the enhancement factor is observed at $d > 0\ \mu m$, nevertheless, for $d = 0.5\mu m$ the modulation in the THz spectral content is practically negligible (blue curve).

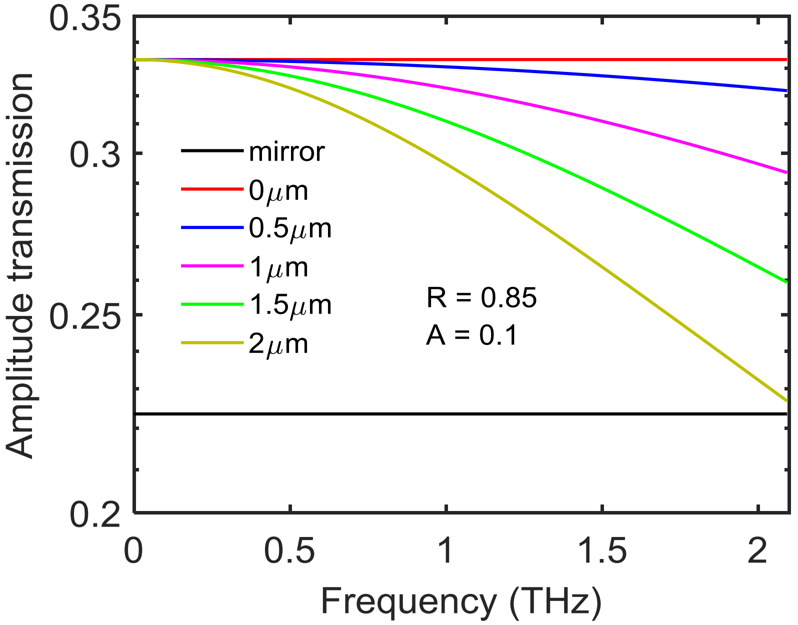

Figure S3: Amplitude transmission of the FP cavity calculated for several cavity lengths $(0\mu m \leq d \leq 2\ \mu m)$ using the FP transfer function (Eq. (1) in the main text file). The FP cavity consists of identical mirrors with parameters R=0.85 and A=0.1. The transmission amplitude of the mirror is shown by the black solid line.

Table S1. Experimentally obtained parameter $r_{m1}r_{m2}$ after fitting the FP cavity transfer function (Eqn. (1)) to the cavity transmittance at different cavity lengths for the single mirror pair m_1-m_2

Cavity length (µm)	Fitted parameters ($r_{m1}r_{m2}$)	
305	0.8294	Mean ($r_{m1}r_{m2}$): 0.83826<br><br>Standard deviation : 0.00615
294	0.8358	
284	0.8349	
272	0.8286	
260	0.8300	
249	0.8340	
237	0.8409	
226	0.8308	
215	0.8346	
204	0.8337	
194	0.8432	
185	0.8361	
175	0.8416	
166	0.8491	
158	0.8489	
149	0.8493	
142	0.8426	
133	0.8407	
126	0.8361	
118	0.8361	
109	0.8355	
101	0.843	
93	0.8409	

Section S4: Phase shift at the reflection of air-mirror interface

In general, the phase shift of a field reflected from the air-metal interface may differ from π, owing to the absorption loss in metallic mirrors. In this section and in section S5 we show that for our experimental parameters, the distortion of the phase from π is practically negligible. This validates our choice to treat the reflection as a real valued coefficient as done throughout the main text. In order to calculate the phase of the reflected field we used the complex refractive index reported previously for a 1.5nm thick Au thin film and for 8nm thick Au film [ref. 1]. We have digitized the real (Fig. 3c in ref.1) and imaginary part (Fig.4c in ref.1) to obtain the frequency dependent complex refractive index of the two samples

The complex reflection coefficient is calculated using the expression outlined in ref. [2] :

$$\tilde{r} = \frac{r_{01}+r_{12}e^{-i\frac{4\pi n_1 d}{\lambda}}}{1+r_{01}r_{12}e^{-i\frac{4\pi n_1 d}{\lambda}}} \quad \text{(s1)}$$

Where r_{01}, r_{12} is the Fresnel reflection coefficient of the air-film and film-quartz interfaces respecitvely, given by $r_{ij} = \frac{n_j - n_i}{n_j - n_i}$, with $n_0 = 1$ (air) is the refractive index of air, $n_1 = n_{Au} - ik_{Au}$ (thin Au film) and $n_2 = 1.955$ is the refractive index of the fused quartz substrate (Fig. S5 in the supplementary information of ref 3]. *d* is the Au film thickness. Figure S4 shows the phase calculated from the reflection coefficient of expression (s1) for the case of 1.5nm Au film (blue curve) and 8nm Au film (red curve).

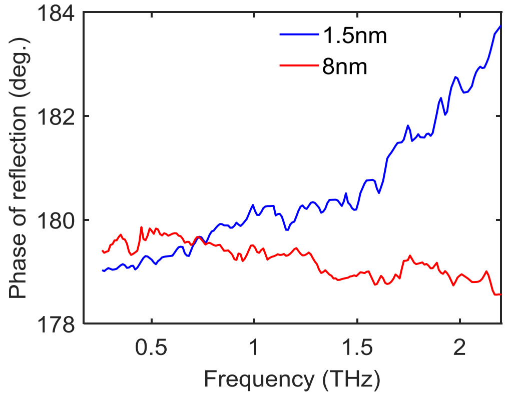

Figure S4 : the reflection phase calculated for our Au-on-fused quartz substrates of 5nm thickness using the complex refractive index extracted from ref.1 for 1.5nm Au film (Blue curve) and for 8nm Au film (Red curve).

Thus for our 5nm Au film (as quantified in section S7), the phase shift of our sample is bound by the blue and red curves, namely in the range of $[178°, 183°]$. We conclude that the maximal phase shift of our reflected field is $< 4°$ for the entire usable bandwidth and $\leq 1°$ for frequencies lower than 1.5THz, validating its negligible distortion from π and our choice to attribute a real-valued reflection coefficient throughout the text.

Section S5: Phase distortion obtained from cavity transmission measurements

In order to further validate the negligibility of the phase shift due to reflection from the air-mirror interface, we returned to our experimental measurements. Here we rely on our ability to monitor multiple FP orders at a fixed cavity length supported by our several octaves spanning THz spectrum. For example, see the blue curve in Fig.2b (with $d = 294\ \mu m$) where we resolve the first 4 resonant frequencies of orders m=1,2,3,4. Those are just an harmonic series of the lowest frequency, and as long as the additional phase concerned here is 0 (i.e. the reflected phase is purely π), the resonance frequencies are equally spaced from one another. Once we include an additional phase in the calculation (a phase that emanates from absorption loss of the Au film), the cavity modes gradually shift and become non-equally spaced anymore.

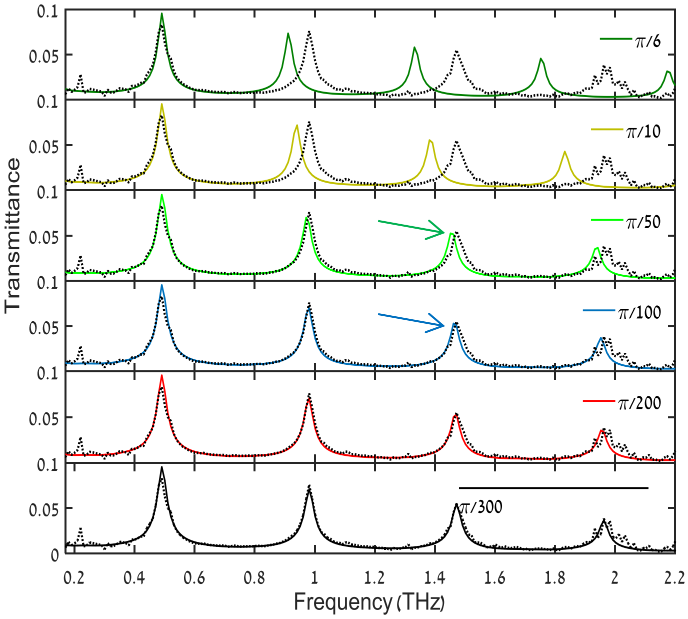

Figure S5: Comparison of the experimental data (black dashed curves) with calculated FP transmission where we 'inject' varying additional phases (ψ) noted in each panel.

The calculation is performed using:

$$T_{FP}(\omega) = \left|\frac{t_1 \cdot t_2 \cdot \exp(-i\varphi(\omega))}{1-|r_{m1}|\cdot|r_{m2}|\cdot\exp(-2i(\varphi(\omega)+\psi))}\right|$$

By comparing the experimental results with the calculated transmission curves we extract an upper limit for ψ , relying on our spectral experimental accuracy to detect a phase shift as small as $\psi = \pi/50$ as readily observed by difference at ~1.5THz (marked by an arrow). For $\psi = \pi/100$ we can hardly resolve the difference between the experimental and calculated peak at ~1.5THz, while the difference at ~2THz is still reasonably observed (despite the noise). We therefore conclude that the additional phase shift imparted by our mirrors is smaller than $\pi/50$, providing clear experimental justification for neglecting this phase and correspondingly treating the reflection coefficients as real-valued throughout the main text file.

Section S6: THz reflection measurement

In order to further verify the quality of our home-made mirrors, we've measured their frequency dependent reflectivity using the THz-TDS setup in reflection configuration shown in Fig.S6(a). Here the angle of incidence is dictated by the off-axis parabolic reflectors at 15^0 to the sample normal. The reflectance of our mirrors is quantified with respect to that of a commercially available gold mirror (PF10-03-M01,Thorlab).

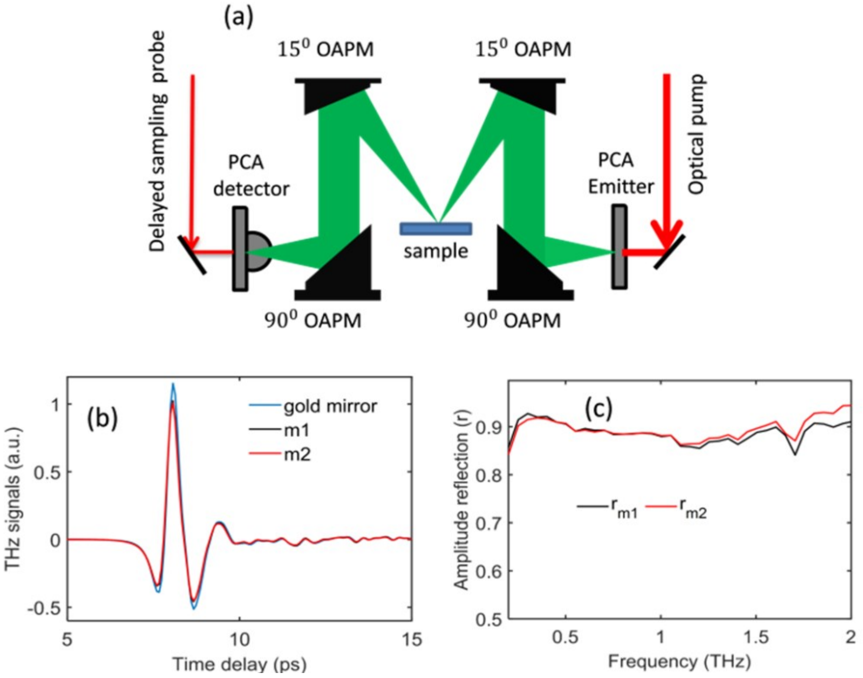

Figure S6: (a) schematic sketch of the THz-TDS reflection setup. (b) The time-domain THz field reflected off the mirrors (m1 and m2) and off the reference reflector (gold mirror, PF10-03-M01 from Thorlabs). (c) Frequency resolved amplitude reflection of mirrors m_1 and m_2.

The schematic of the THz setup in the reflection geometry is shown in Fig. S6(a). The THz field is routed by four off-axis parabolic mirrors (OAPM). The first 90^0 OAPM collimates the THz beam and the second 15^0 OAPM focuses the THz beam onto the sample surface. The THz field reflected from the sample is collected and collimated by the third 15^0 OAPM and finally focused by the last 90^0 OAPM onto the PCA detector.

As can be readily observed in time domains signal of Fig.S6(b) the THz field reflected from the mirror is attenuated to about 90% from of that of the reference, with similar temporal shape. The frequency-dependent reflectance coefficient (r_{mi}) is obtained by $|r_{mi}| = \left|\frac{E_{mi}}{E_{gold-mirror}}\right|$, is shown in Fig. S6(c) with no apparent modulations across the usable THz spectrum, i.e. showing a 'flat' response as expected from homogeneous metallic surface.

Section S7: Atomic Force Microscopy image of representative mirror

The morphology of the mirrors surface was characterized by atomic force microscope (AFM) operating at room temperature in a contact mode and the images were analyzed using the Gwyddion software. The film thickness was measured by the height difference across a designated step created by scratching the film surface with a sharp razor and scanning across the step as shown in Fig S7 (a). The white line illustrates the scanning segment across the designated step and the corresponding height profile is shown in Fig. S7 (b). The film height is 5nm. In order to quantify the surface morphology we have scanned an area of 0.5μm x 0.5μm shown in Fig. S7 (c) which exhibits a uniform and continuous surface with surface roughness less than 1nm. The roughness profile is shown in Fig. S7 (d).

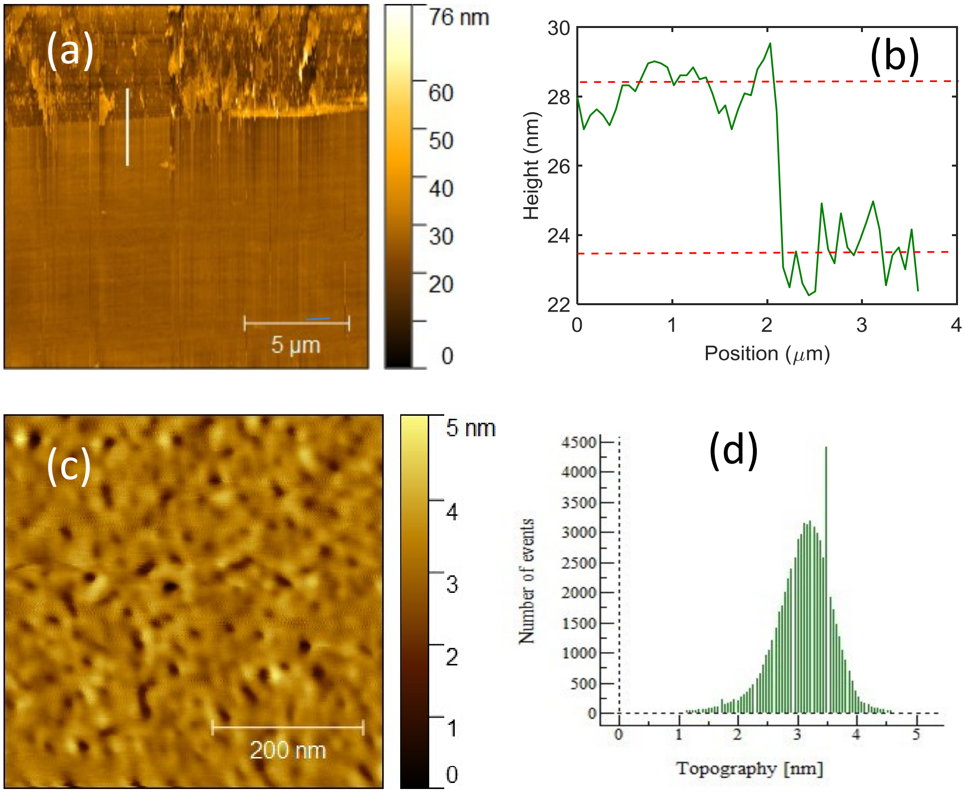

Figure S7: (a) AFM height segment (b) height profile (c) AFM image showing the surface morphology of the mirror. (d) Roughness profile with mean square roughness of 0.6nm.

References

1 Zhou D, Parrott EPJ, Paul DJ, Zeitler JA. Determination of complex refractive index of thin metal films from terahertz time-domain spectroscopy. *J Appl Phys* 2008; **104**: 53110.

2 Bae J, Park J, Ahn H, Jin J. Optical method for simultaneous thickness measurements of two layers with a significant thickness difference. *Opt Express* 2021; **29**: 31615.

3 Zhou Y, E Y, Ren Z, Fan H, Xu X, Zheng X *et al.* Solution-processable reduced graphene oxide films as broadband terahertz wave impedance matching layers. *J Mater Chem C* 2015; **3**: 2548–2556.
